# Effect of recipe composition and storage conditions on *Salmonella* survival in sauces made from contaminated tahini paste

**DOI:** 10.1128/spectrum.00520-26

**Published:** 2026-05-28

**Authors:** Mary Rao, Sandeep Tamber

**Affiliations:** 1 Bureau of Microbial and Toxicological Food Safety, Health Canada, Ottawa, Ontario, Canada; University of Mississippi, University, Mississippi, USA

**Keywords:** *Salmonella enterica*, foodborne illness, low-moisture foods, sesame, water activity, acidification, refrigeration

## Abstract

Tahini paste, a low-moisture food, has been implicated in 20 salmonellosis outbreaks since 1995, with over 1,600 reported cases of illness. When used to prepare high-moisture foods (e.g., tahini sauce, hummus), contaminated tahini paste can pose an increased risk to consumers due to potential bacterial growth. This study examined three naturally contaminated tahini samples to assess microbial load and evaluated the impact of preparation and storage practices on *Salmonella* replication in tahini sauces made from tahini paste. Naturally contaminated tahini paste harbored low concentrations of *Salmonella* (~0.4 most probable number [MPN]/100 g), with no correlation to background microbiota. Tahini sauces made with artificially contaminated tahini paste and various diluents (water, oil, and lemon juice) with and without garlic powder showed variable *Salmonella* recovery at 22°C and 4°C. *Salmonella* levels in water-based sauces (a_w_ = 0.9969) increased by 6 logs over 24 h at 22°C. In contrast, levels in oil-based sauces were stable at 2 log CFU/g (a_w_ = 0.1918). Sauces prepared with lemon juice had reduced levels of *Salmonella* recovery, with fewer cells recovered at higher concentrations of lemon juice (<1 log CFU/g with 50% lemon juice [vol/vol], pH 3.6). At 4°C, *Salmonella* levels in both water- and oil-based sauces remained stable for two weeks at 2 log CFU/g, whereas no *Salmonella* were recovered from lemon juice-based sauce (50% vol/vol) by one week of storage. Garlic powder (0.7%, wt/vol) did not affect *Salmonella* recovery. These findings support the use of lemon juice and refrigeration by end-users as effective interventions to help mitigate *Salmonella* risk in tahini sauces.

**IMPORTANCE:**

Tahini paste, a seed butter made from sesame seeds, has been implicated in numerous salmonellosis illnesses and product recalls. The use of contaminated tahini paste as an ingredient in ready-to-eat sauces and dips such as hummus poses a risk to human health. In order to understand these risks and to provide guidance to consumers, this study examined the influence of recipe ingredients and storage conditions on the growth of *Salmonella* in sauces prepared with contaminated tahini. Ingredient choice, particularly the incorporation of an acidulant such as lemon juice, had a strong negative impact on *Salmonella* replication, while refrigerated storage of prepared sauces further reduced bacterial recovery. These findings offer practical strategies for consumers and food service professionals to reduce *Salmonella* risk and enhance the food safety of products made with tahini paste.

## INTRODUCTION

*Salmonella enterica* is a leading cause of bacterial foodborne illness. Infections are typically associated with consumption of foods originating from animals such as meat, poultry, and eggs (1). However, recently a number of large outbreaks have been linked to non-meat foods (2, 3). Of particular concern are outbreaks associated with the consumption of low-moisture foods. These foods are defined as having water activities (a_w_) of 0.85 or less and include dried foods, powders, spices, chocolate, and nut and seed butters. The lack of available water in low-moisture foods prevents microbial growth; however, several bacterial species, including *Salmonella enterica,* can survive for years in these foods (4).

Tahini paste, a staple of Middle Eastern cuisine, is a seed butter made from ground sesame seeds. When mixed with other ingredients such as water, lemon juice, and spices such as garlic, it forms the basis of many sauces, dips, and condiments (5). It is a low moisture, shelf-stable food with an average water activity of approximately 0.16 and moisture contents ranging from <1% to 1.5% (6, 7).

There are multiple routes for *Salmonella* contamination of tahini paste. Sesame seeds can be contaminated by *Salmonella* strains present in growing areas. Bacteria may also be introduced via cross-contamination at multiple steps during processing (5). Sesame seeds are typically roasted prior to grinding. While roasting parameters likely vary among producers, roasting at a minimum of 110°C for 60 min was sufficient for a five-log reduction of *Salmonella* (8). Given its manufacturing process and low water activity, tahini is considered a ready-to-use food with a long shelf-life. However, should any *Salmonella* cells survive the roasting process or if some are introduced after roasting, *Salmonella* can survive in tahini paste for at least 16 weeks (8, 9). This long-term persistence emphasizes the need for validated roasting procedures and strict adherence to hygienic food manufacturing principles.

Tahini paste has a global distribution, and imported products have been implicated in multiple salmonellosis outbreaks around the world (5). Many of these outbreaks have been linked to the consumption of higher-water-activity foods, such as hummus, which were prepared locally using contaminated imported tahini paste (5). Foods with a minimum water activity of 0.93 can support the growth of *Salmonella*, and higher doses of the pathogen increases the likelihood and severity of illness (10). Therefore, the presence of contaminated tahini paste in home kitchens presents a food safety hazard to consumers. There is considerable information on pathogen reduction interventions that can be applied to tahini paste at the large-scale or producer level, but there is little data to support the development of accessible guidance to consumers (5, 11, 12). To better understand the risks naturally contaminated tahini paste can pose to end-users, our aims were to determine the levels of *Salmonella* associated with the product and examine how bacterial levels can change during small-scale production of tahini sauce from contaminated tahini paste. Specifically, we determined the impact of recipe ingredients, their quantities, and different storage conditions on *Salmonella* levels. The results of this work can be used to improve food hygiene and help reduce the likelihood of illnesses resulting from the ingestion of contaminated tahini paste.

## MATERIALS AND METHODS

### Food samples

Three tahini paste samples naturally contaminated with *Salmonella* were used in this study. Two samples of recalled product were obtained from the Canadian Food Inspection Agency (CFIA). These samples were identified as positive through the testing activities of the CFIA and were in their original retail packages. The third sample was donated to the study after being identified as a recalled product. This sample was unopened and in its original retail packaging. Negative tahini paste samples used for artificial contamination were purchased at retail. Vegetable oil, bottled shelf-stable lemon juice (Ingredients: water, concentrated lemon juice, sulphites, lemon oil; contains 4.65% citric acid), and garlic powder were purchased at retail. All food samples were stored at room temperature (22°C) in their original packaging for the duration of the study.

### Bacterial enumeration by direct plating

Levels of background microbiota were determined using standard plate count methodology. Five 25 g samples of naturally contaminated tahini paste were serially diluted 10-fold in buffered peptone water (BPW; Becton Dickinson, Fisher Scientific, Ottawa, ON) and plated in duplicate on plate count agar [total aerobic mesophiles, TAM], *Enterobacteriaceae* Petrifilm (EB, Innovation Diagnostics), and coliform Petrifilm (CF, Innovation Diagnostics). Plates were incubated at 35°C for 24 h.

### *Salmonella* enumeration by the MPN method

A three-dilution, five-tube most probable number (MPN) analysis was used to determine *Salmonella* levels in the naturally contaminated tahini paste. Each MPN tube was tested for the presence of *Salmonella* using the Canadian reference method for this pathogen (MFHPB-20) (13). Briefly, 900 mL BPW was added to 100 g aliquots, which were then mixed for 2 min at high speed in a circulator lab blender (Seward Stomacher, Fisher Scientific, Ottawa, ON). Samples were incubated overnight at 35°C. Selective enrichment was carried out in Rappaport-Vassiliadis *Salmonella* and Tetrathionate-Brilliant-Green broths for 24 h at 42.5°C. Cultures were streaked onto xylose lysine deoxycholate (XLD) and brilliant green sulfa agars. The plates were incubated at 35°C for 24 h and 48 h and analyzed for the presence of *Salmonella*-like colonies. The identity of at least three colonies of distinct morphology per sample type was confirmed by serology, biochemical reactions on MacConkey agar, triple sugar iron agar, lysine iron agar, testing for the presence of the *invA* gene (14), and MALDI-TOF (15). MPN values were calculated according to the method of Garthright and Blodgett (16). The serovar of the recovered isolates was determined using *Salmonella* antisera according to the Kauffmann-White-Le Minor scheme (17). All media and antisera used for the MPN determination were manufactured by Becton Dickinson and obtained from Fisher Scientific, Ottawa, ON.

### Analysis of tahini sauce prepared from naturally contaminated tahini paste

A basic tahini sauce was prepared using naturally contaminated tahini paste (250 g) and sterile water (250 mL). These two ingredients were added to a large laboratory sampling bag (Whirl-Pak, Thermo Fisher Scientific) and mixed by first massaging the bag by hand and then mixing in a circulator lab blender for 2 min at high speed. The tahini sauce was divided into two aliquots of equal weight. One was incubated at ambient temperature (22°C), and the other at 4°C for 6 h. After 6 h, the tahini sauces were analyzed for the presence of *Salmonella* using MFHPB-20 (previous section). Five 25 g aliquots of each were also taken for enumeration of *Enterobacteriaceae* by direct plating as described previously.

### Inoculation of tahini paste

Tahini paste was purchased at retail and confirmed to be negative for *Salmonella* by MFHPB-20. Five *S*. *enterica* strains belonging to serovars previously implicated in tahini-related outbreaks or recalls (5) were selected from the laboratory’s culture collection, which contains *Salmonella* strains isolated from diverse food sources in Canada over a span of 40 years. When a tahini isolate was not available, one from a low-moisture source was chosen. The serovars and isolation sources are as follows: Liverpool (sesame), Montevideo (tahini), Orion (tahini), Havana (animal feed), and Mbandaka (tahini). The exact dates of the original isolation are not known but are believed to have been before 1983. Strains were routinely retrieved from storage at −80°C by plating onto tryptic soy agar (TSA) and incubating overnight at 35°C. Overnight cultures were prepared in tryptic soy broth and collected after incubation at 35°C with shaking at 250 rpm for 24 h. Cells were harvested by high-speed centrifugation (13,000 rpm × 1 min) and washed once in phosphate-buffered saline (PBS). Cell pellets were resuspended in PBS and diluted to achieve a cell concentration of 10^7^ CFU/mL. A 5-strain cocktail was prepared by combining 200 µL of each strain suspension per 1 mL cocktail. For each biological replicate, a bulk sample of tahini paste was inoculated to a target inoculum of 10^4^ CFU/g by adding 100 µL strain cocktail per 25 g tahini paste in a laboratory sampling bag and mixed by massaging by hand for 30 s. Inoculated samples were stored in the dark at room temperature (22°C) for 14 days to allow for bacterial acclimation.

### Tahini sauce preparation and characterization

Tahini sauces were prepared using tahini paste that had been inoculated with the 5-strain *Salmonella* cocktail and acclimated for 14 days at 22°C. To obtain a recipe for tahini sauce, an internet search using “tahini sauce recipe” was conducted to obtain information on how tahini sauces are prepared (Supplementary Information, Tables S1 and S2). Using the first fifteen recipes from the internet search as a guide, tahini sauces were prepared in bulk in laboratory sampling bags by adding the appropriate volume of water, oil, and lemon juice as indicated in Table 3. Garlic powder, when required, was added at a concentration of 1 teaspoon (or 2 g) per 300 g sauce. For each biological replicate, tahini sauces were mixed until smooth, portioned into individual 25 g samples, and incubated at room (22°C) or refrigerated temperatures (4°C) for the stated times.

The consistency of the sauces was assessed qualitatively based on appearance and ability to be aspirated with a pipet. Water activity (a_w_) was determined using 3 g portions of tahini sauce and a dew point water activity meter (AquaLab 4TE, Decagon Devices). Water activity results reported are averages of two readings taken from each sample. pH readings were determined in duplicate using 20 mL portions of two independently prepared sauce samples and a combination pH electrode (Orion Ross Ultra, Thermo Scientific). All measurements were conducted with samples at room temperature (22°C).

### *Salmonella* recovery and enumeration from inoculated tahini paste and tahini sauces

Two hundred twenty-five millilitres of BPW was added to each 25 g tahini paste or tahini sauce sample. Samples were mixed at high speed for 2 min in a circulating laboratory blender, diluted serially in BPW, and plated in triplicate on xylose lysine deoxycholate agar. The remainder of the sample-BPW mixture was incubated overnight at 35°C and analyzed for the presence of *Salmonella* as described in MFHPB-20.

### Inhibition of *S. enterica* by lemon juice

The inhibitory concentration of retail lemon juice against the five *S*. *enterica* strains was assessed using a broth microdilution method. Two-fold serial dilutions of lemon juice were prepared in a round-bottom 96-well plate using Luria broth (LB) as the diluent (50 µL lemon juice:50 µL LB). Freshly plated strains were taken from TSA and diluted in saline to achieve a cell density corresponding to a McFarland standard of 0.5. These cultures were diluted 100-fold in LB, and 50 µL was added to the dilution plate. The final concentration of lemon juice assessed was 50%–0.05%. Inoculated plates were incubated at 35°C for 18 h and scored visually for growth. The experiment was repeated twice using independent cultures, and the results reported are the mode of 10 values.

### Data analysis and statistics

Results reported are the mean values plus or minus their standard deviation of three biological replicates using independent cultures, freshly inoculated tahini, and freshly prepared sauces with three technical replicates each. The limit of quantitation by direct plating was 1 log CFU/g. Replicates in which no cells were recovered by direct plating were considered zero for the purposes of statistical calculation. Differences between means were calculated by Student’s *t*-test or ordinary one-way ANOVA with Tukey’s test for multiple comparisons as appropriate. Comparisons with adjusted *P* values of less than 0.05 were considered significantly different. Statistical calculations were conducted with GraphPad Prism version 9.

## RESULTS

### Background microbiota levels in naturally contaminated tahini paste

The levels of three bacterial groups, total aerobic mesophiles (TAM), *Enterobacteriaceae* (EB), and total coliforms (CF), found in naturally contaminated tahini paste are shown in Table 1. The TAM level was similar among the three samples, approximating log 3 CFU/g. The EB and CF levels approached 2 log CFU/g in all three samples, with tahini paste sample three having a significantly lower EB concentration than tahini paste samples 1 and 2 (*P* < 0.05).

**TABLE 1:** Background microbiota associated with naturally contaminated tahini

Sample	Total aerobic mesophiles	*Enterobacteriaceae*	Coliforms
Tahini 1	2.9 ± 0.1 log CFU/g	2.4 ± 0.2 log CFU/g	1.9 ± 0.3 log CFU/g
Tahini 2	3.0 ± 0.1 log CFU/g	2.4 ± 0.2 log CFU/g	1.8 ± 0.3 log CFU/g
Tahini 3	2.8 ± 0.2 log CFU/g	1.9 ± 0.3^*a*^ log CFU/g	1.5 ± 0.2 log CFU/g

^*a*^ Significantly lower than tahini samples 1 and 2 (*P* < 0.05).

### *Salmonella* levels in naturally contaminated tahini paste

The levels of *Salmonella* associated with the three naturally contaminated tahini paste samples were too low to quantify by direct plating. Therefore, an MPN method was used to determine *Salmonella* concentration. Using an analytical unit of 500 g, MPN levels of 0.2 (0.03–1.6), 0.2 (0.03–1.6), and 0.7 (0.2–2.3) per 100 g tahini paste were obtained for samples 1 to 3, respectively (Table 2). These levels translated to one cell per every 500 g (samples 1 and 2) or 143 g (sample 3) of tahini. One serovar was recovered from each tahini sample; Montevideo (sample 1), Menston (sample 2), and Kaevlinge (sample 3).

**TABLE 2:** *Salmonella* concentration of naturally contaminated tahini

Sample	MPN tube reading	MPN/100g (LCL-UCL)^*a*^	Serogroup	Serovar
Tahini 1	1-0-0	0.2 (0.03–1.6)	O:7 (C1)	Montevideo
Tahini 2	1-0-0	0.2 (0.03–1.6)	O:7 (C1)	Menston
Tahini 3	1-1-1	0.7 (0.2–2.3)	O:16 (I)	Kaevlinge

^*a*^ Lower and upper 95% confidence limits.

### Recovery of bacteria from tahini sauce prepared with naturally contaminated tahini paste

**Fig 1:**
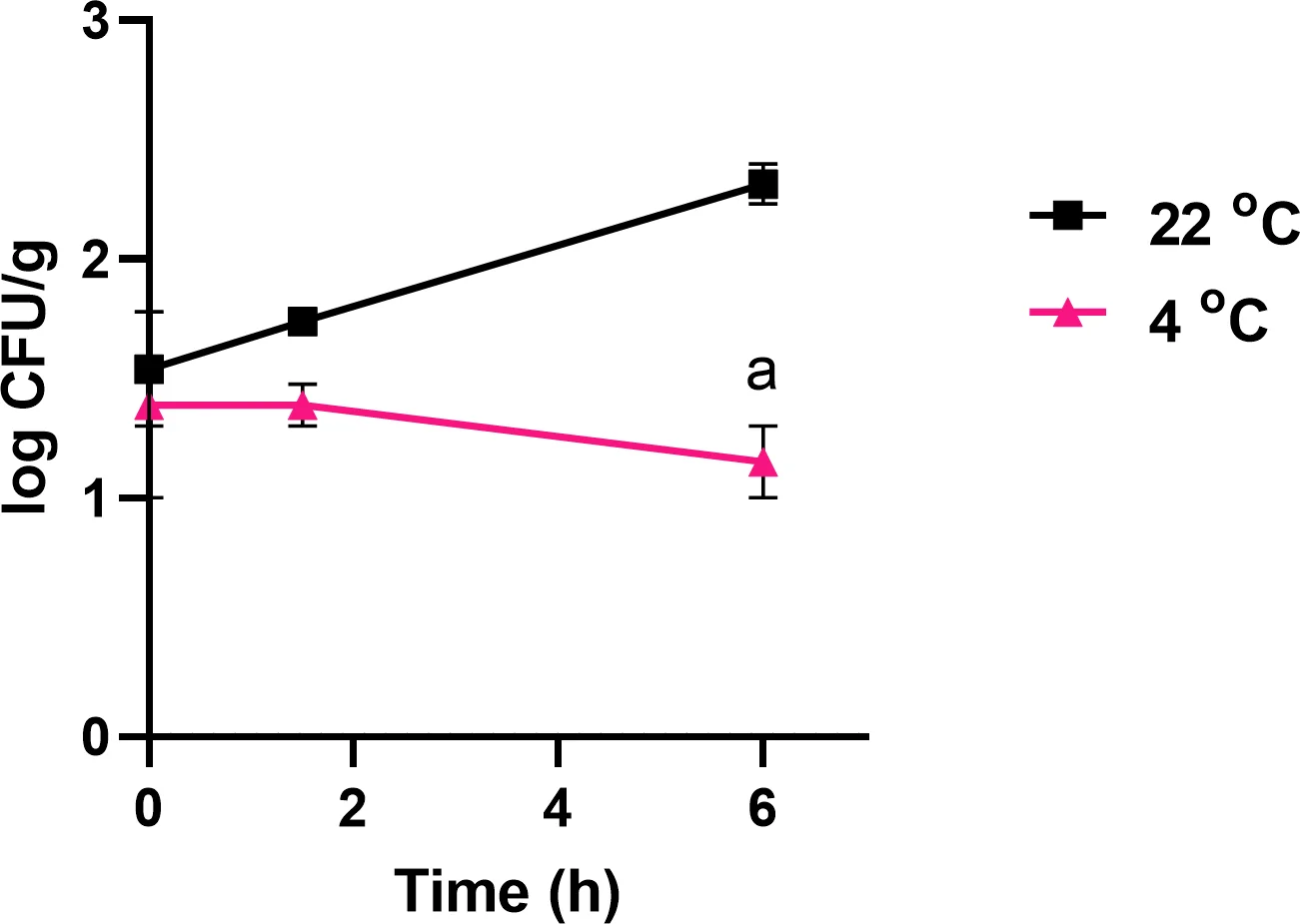
Change in *Enterobacteriaceae* numbers in prepared tahini sauce. Tahini paste was diluted 1:1 with water and incubated at room temperature (22°C) or refrigeration (4°C) temperatures. *Enterobacteriaceae* counts were determined at the indicated time points. ^a^Counts at 6 h were significantly higher at ambient temperature than at refrigerated temperatures, as assessed by Student’s *t*-test.

To assess the potential for bacterial growth when contaminated tahini paste is used for food preparation, a basic tahini sauce was prepared from the contaminated product. Half of the prepared sauce was stored at room temperature (22°C), and the other half was refrigerated (4°C). After 6 h of storage at room temperature, the *Enterobacteriaceae* levels increased from 1.5 log CFU/g to 2.3 log CFU/g. No increase was observed in the refrigerated sauce, and significantly fewer cells were recovered after 6 h of refrigerated storage than room temperature (1.2 log CFU/g vs 2.3 log CFU/g, respectively) (Fig. 1, *P* < 0.05). No *Salmonella* was detected at the 6 h time point in either sauce by the MPN method.

### Recovery of *Salmonella* from tahini sauces prepared with artificially contaminated tahini paste

A retail sample of tahini paste was inoculated with a five-strain *Salmonella* cocktail and stored at room temperature (22°C). Over 14 days, the initial *Salmonella* inoculum decreased from 3.6 log CFU/g to 1.1 log CFU/g. The majority of the decline (0.9 log CFU/g) occurred within the first 3 days, and levels appeared to plateau by the end of 2 weeks. (Fig. S1). Subsequent portions of tahini paste were inoculated and stored for 14 days at room temperature prior to tahini sauce preparation to allow for pathogen acclimation.

**Fig 2:**
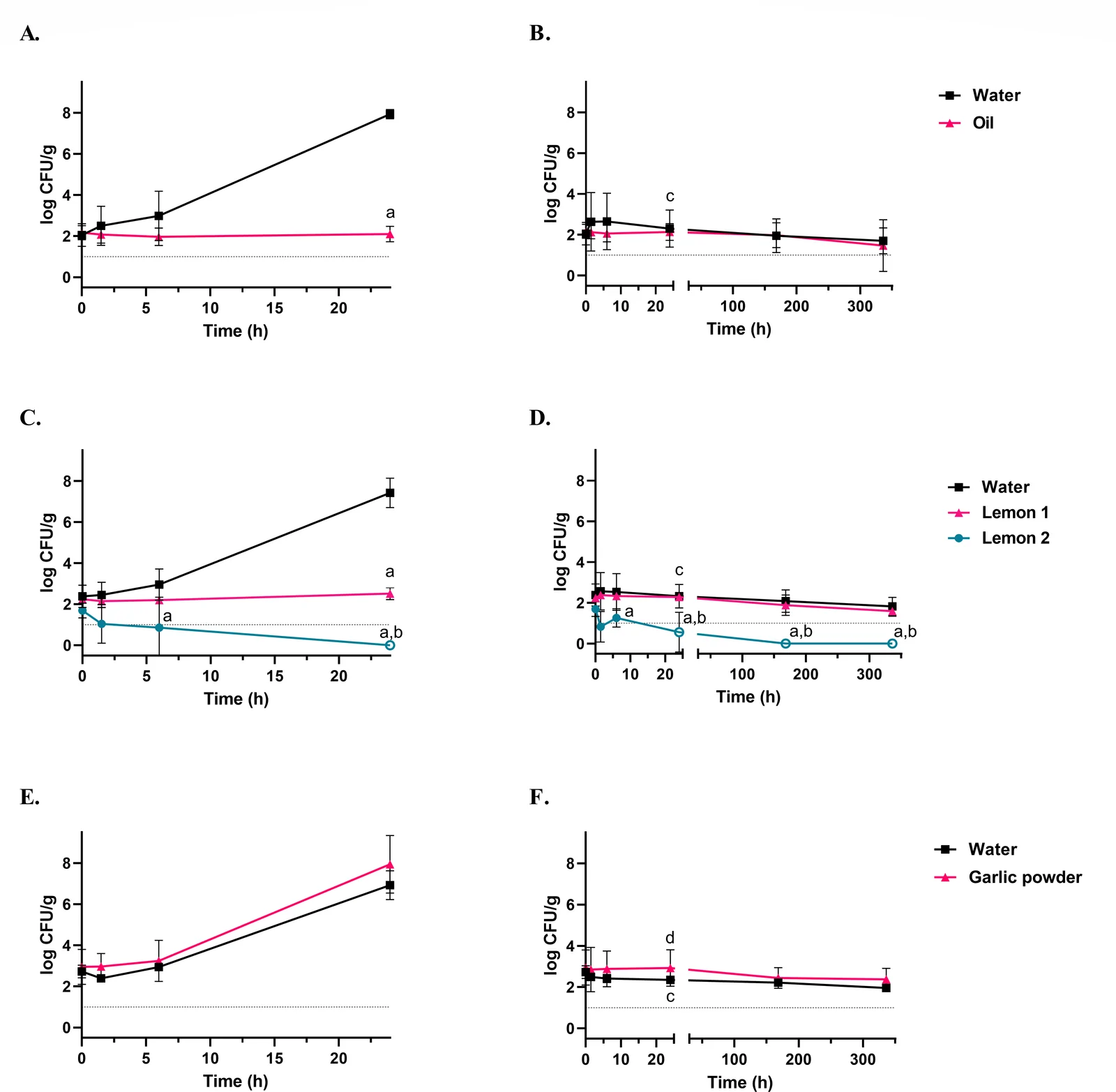
Recovery of *S. enterica* from tahini sauces made from artificially contaminated tahini paste. 2A and 2B. Recovery from water-based tahini sauce (black) and oil-based tahini sauce (pink) at room (22°C; **A**) or refrigerated (4°C; **B**) temperatures. 2C and 2D. Recovery from water-based tahini sauce (black), Lemon 1 (25% lemon juice, pink), and Lemon 2 (50% lemon juice, blue) at room (22°C; **C**) or refrigerated temperatures (4°C; **D**). Open symbols indicate time points where viable *Salmonella* was not recovered by enrichment in at least one replicate. 2E and 2F. Recovery from water-based tahini sauces without (black) and with added garlic powder (pink) at room temperature (22°C; **E**) or refrigerated temperatures (4°C; **F**). Lowercase letters indicate statistically significant differences at a significance level of *P* < 0.05. a = conditions where recovery was lower than that of the water-based tahini sauce; b = conditions where recovery was lower than that of the Lemon 1 tahini sauce; c and d = conditions where recovery from water-based tahini sauce (**C**) and tahini sauce containing garlic powder (**D**) was lower at refrigerated temperature than at room temperature (22°C vs 4°C). Horizontal gray dash indicates the limit of quantitation by direct plating (1 log CFU/g).

To determine if the types and quantities of ingredients used to make tahini sauce could impact *Salmonella* levels, artificially inoculated tahini paste was used to make various tahini sauces (Table 3). Taken from the jar, tahini paste is a viscous material that is difficult to manipulate. Adding liquid made the mixture seize (clump), but a fluid consistency amenable to pipetting was obtained when equal volumes of diluent were added to the tahini paste. The levels of *Salmonella* recovered from a basic tahini sauce consisting of tahini paste and water mixed at a 1:1 ratio (a_w_ = 0.9969, pH = 5.9) and stored at room temperature (22°C) for 24 h increased from 2.1 log CFU/g to 7.9 log CFU/g. In contrast, when a tahini sauce was prepared with vegetable oil and stored at 22°C (1:1 mixture, a_w_ = 0.1918), the *Salmonella* levels remained steady at approximately 2 log CFU/g for all time points (Fig. 2A).

**TABLE 3:** Tahini sauce recipes and properties

Sauce	Ingredients	a_w_	Lemon Juice and (citric acid) concentration	pH
Water	1:1^*a*^ tahini paste and water	0.9969	NA^*b*^	5.9 ± 0.1
Oil	1:1 tahini paste and vegetable oil	0.1918	NA	NA
Lemon 1	2:1:1 tahini paste, water, and lemon juice^*c*^	0.9892	25% (1.2%)	4.2 ± 0.1
Lemon 2	1:1 tahini paste and lemon juice	0.9851	50% (2.3%)	3.6 ± 0.1
Garlic Powder	1:1 tahini paste, water, and two g^*d*^ garlic powder per 300 g sauce	NA	NA	NA

^*a*^ Ratios are by weight.

^*b*^ N/A, not applicable.

^*c*^ Shelf-stable lemon juice made from concentrate, purchased at retail.

^*d*^ 2 g is approximately one teaspoon.

An internet search (Table S1) revealed many tahini sauce recipes use tahini paste, water, lemon juice, and garlic as the main ingredients, with varying quantities and shelf lives reported. Lemon juice concentrations in the final tahini sauces ranged from 13% to 38% (average, 23 ± 9% vol/vol). A tahini sauce prepared to contain a final lemon juice concentration of 25% (Lemon 1, Table 3) resulted in stable levels of *Salmonella* (~2 log CFU/g) over a 24 h holding period at room temperature (Fig. 2B). These levels were significantly reduced compared with those of the water-based tahini sauce at 24 h (6.9 log CFU/g vs 2.5 log CFU/g, *P* < 0.05). When the lemon juice concentration was doubled to 50% [vol/vol] (Lemon 2, Table 3), further log reductions of *Salmonella* were observed. After 90 min of storage at room temperature, the level of *Salmonella* in the Lemon 2 tahini sauce decreased from 1.7 log CFU/g to 1.0 log CFU/g. By 6 h, this number declined to 0.9 log CFU/g, and no cells were recovered by direct plating at 24 h (significantly lower than that of the water-based sauce at both time points and significantly lower than that of Lemon 1 at 24 h; *P* < 0.05). Enrichment of the 24 h samples resulted in the recovery of *Salmonella* in 1 of the 3 biological replicates.

The number of garlic cloves reported in the tahini sauce recipes collected from the internet (Table S1) ranged from 0 to 15 per 300 mL tahini sauce (average = 4 ± 4 cloves). Given the variability associated with fresh garlic cloves with respect to size and potency, we opted to use garlic powder to investigate potential impacts on *Salmonella* recovery. Addition of the equivalent of 4 cloves to 300 mL sauce (Table S2) did not impact *Salmonella* levels in comparison with those of the water-based sauce (Fig. 2C), or those of a sauce containing 25% lemon juice (data not shown).

At refrigerated temperatures, the levels of *Salmonella* recovered from the tahini sauces approximated 2 log CFU/g at all time points (Fig. 2B, D, and F). The one exception was the tahini sauce prepared to a final lemon juice concentration of 50% (Lemon 2). In that sauce, the levels of *Salmonella* gradually declined from 1.8 log CFU/g to 1.0 log CFU/g after 6 h, and no cells were recovered by direct plating at subsequent time points. The levels observed in the Lemon 2 sauce were significantly less than those observed in the water-based sauce from 6 h to 2 weeks and lower than those observed in the sauce containing half the amount of lemon juice (Lemon 1) from 24 h to 2 weeks (Fig. 2D, *P* < 0.05). Enrichment of the refrigerated Lemon 2 samples recovered viable *Salmonella* at 24 h (2/3 replicates positive) and 1 week of storage (2/3 replicates positive); no cells were recovered from the 2-week samples.

Compared with storage at room temperature, significantly fewer *Salmonella* cells were recovered from the refrigerated water-based and garlic powder-containing tahini sauces after 24 h of storage (approximately 5 log lower, *P* < 0.05).

### Relationship between lemon juice concentration and *S. enterica* recovery

The minimal inhibitory concentration (MIC) of lemon juice against the five strains of *Salmonella* used in this study was 0.8%, which translated to a citric acid concentration of 0.04%. Using lemon juice to a final concentration of 25% in tahini sauce (Lemon 1, 1.2% citric acid) decreased the pH relative to that of the water-based sauce from 5.9 to 4.2. The pH of the Lemon two sauce (50% lemon juice, 2.4% citric acid) was 3.6. The pH values of the tahini sauces remained stable over 24 h of storage at 22°C.

## DISCUSSION

The presence of *Salmonella* in tahini paste presents a hazard to consumers, particularly given tahini paste’s common use in ready-to-eat sauces and dips. The stability of *Salmonella* in tahini paste combined with a long product shelf life further compounds this risk. We observed very little change in *Salmonella* levels in tahini paste over 2 weeks, and others have reported similar results over months of storage (8, 9). This persistence may result in long-term, repeated exposures to *Salmonella* as consumers use tahini paste in multiple meals over the long shelf life of the product.

The water activity of tahini paste is too low to support bacterial growth. However, when contaminated tahini paste was diluted and stored at room temperature for 6 h, a significant increase in *Enterobacteriaceae* was observed. Supporting this result was the significant (5 log) increase of *Salmonella* observed after diluting artificially contaminated tahini paste and storing it at room temperature overnight. These observations demonstrate the potential for bacteria to grow in diluted tahini sauces if stored improperly. No *Salmonella* growth was observed when prepared tahini sauces were stored in the refrigerator. Using vegetable oil as a diluent or adding ingredients such as lemon juice also limited *Salmonella* replication. In fact, a decrease in *Salmonella* numbers was observed in tahini sauce prepared with lemon juice (50%, vol/vol). Weak acids have long been used as food preservatives. They have multiple points of action, including denaturation of membrane proteins, energy expenditure to maintain the proton motive force (PMF), and eventual loss of the PMF leading to acidification of the cytoplasm (18). Studies using broth cultures and mayonnaise show *Salmonella* levels decline as the pH of the matrix decreases and approaches the pKa of the weak acid in question (19–21). This is supported by our results showing a greater reduction in *Salmonella* levels in the Lemon 2 tahini sauce (50% lemon juice, pH 3.6) than in the Lemon 1 sauce (25% lemon juice, pH 4.2). The Lemon 1 and Lemon 2 tahini sauces contained 31-fold to 63-fold more citric acid than the MIC determined for the commercial lemon juice in aqueous solution. These higher concentrations may be required to penetrate the tahini matrix, which is viscous and high in fats and proteins. Al-Nabulsi *et al*. reported significant reductions of *Salmonella* using 0.5% citric acid in a 10% tahini solution (12). Considering the tahini sauces in this study contained five times more tahini paste, the higher inhibitory citric acid concentration observed here (2.4%) aligns with the requirement for increased acidity in matrices containing more solids.

Compared to water, the addition of garlic powder did not impact the recovery of *Salmonella* from tahini sauce. This result is contrary to other reports and may reflect the decision to use garlic powder rather than fresh garlic (22). The active ingredient in garlic, allicin, has been shown by others to exert an antimicrobial effect on *Salmonella in vitro* and in meat matrices such as mutton, ground beef, and chicken skin (23–25). However, allicin is an unstable compound, and its concentration in garlic is variable. Processing of garlic into a powder has been shown to reduce the efficacy of allicin (26). Future studies should consider the use of fresh garlic to deliver a standard concentration of allicin.

It is important to note that viable *Salmonella* were recovered from all sauce types and storage conditions with the exception of the Lemon 2 tahini sauce (50%, vol/vol) stored for 2 weeks at 4°C. Visually, sauces stored for 1 and 2 weeks in the refrigerator did not differ from freshly prepared sauces. No other sensory analyses were done, and these would be required to determine the palatability of a tahini sauce containing 50% (vol/vol) lemon juice and stored at 4°C for 2 weeks. Although taste is subjective, none of the recipes collected as part of this study recommended a sauce containing 50% lemon juice. The majority suggested two parts tahini paste to one part lemon juice. Therefore, future experiments may consider the use of herbs and spices such as thyme, cumin, and mustard that have documented activity against *Salmonella* to act as additional hurdles in tahini sauces (27, 28).

The levels of *Salmonella* detected in the contaminated tahini pastes were low, averaging one cell per 273 g tahini paste. These levels were consistent with previously reported values associated with human illness (Paine et al., 2014; Unicomb et al., 2005). Given these low levels, complete absence from tahini paste and subsequent sauces would be required to assure consumer safety. The levels of background microbiota associated with the three naturally contaminated tahini samples were also low, despite the presence of *Salmonella*. While enumeration of total aerobic mesophiles, *Enterobacteriaceae*, or coliforms is valuable for assessing process control, it does not ensure the absence of *Salmonella*. Based on our results, end-point testing using sample sizes in excess of 250 g would allow for more reliable verification of the absence of *Salmonella* in these products. However, end-product testing alone is not sufficient to ensure product safety. Rather, it is critical to maintain hygiene from the handling of incoming sesame seeds through every stage of the tahini manufacturing process. Adherence to good manufacturing and hygienic principles as laid out by the Codex Alimentarius Commission and the Grocery Manufacturers Association of the United States can help minimize the presence of *Salmonella* in tahini (29, 30).

### Conclusions

The presence of *Salmonella* in tahini paste has been an ongoing public health issue for over 30 years. Tahini paste can become contaminated from multiple sources both pre- and postharvest. Once contaminated, tahini paste can sustain *Salmonella* for prolonged periods. The low levels of *Salmonella* found in tahini paste necessitate larger sample sizes ( >250 g) for reliable detection. Our data show interventions can be applied when preparing tahini sauces that can reduce the rate of *Salmonella* replication should it be present. These interventions include preparing sauces with commercial lemon juice and storing prepared sauces in the refrigerator. These measures, however, should be considered an additional layer of protection, as they will not eliminate the risk of salmonellosis should contaminated sauces be consumed. Therefore, effective management of the tahini production process by using sesame seeds free of *Salmonella* and maintaining strict hygiene over all aspects of the manufacturing process will ensure consumer safety.

## SUPPLEMENTAL MATERIAL

Supplemental tables and figure spectrum.00520-26-s0001.xlsx Tables S1 and S2, and Figure S1.

ASM does not own the copyrights to Supplemental Material that may be linked to, or accessed through, an article. The authors have granted ASM a non-exclusive, world-wide license to publish the Supplemental Material files. Please contact the corresponding author directly for reuse.
